# The characteristic “alveolus nest sign” in solid pseudopapillary neoplasm of the pancreas

**DOI:** 10.1055/a-2353-6114

**Published:** 2024-07-29

**Authors:** Weigang Gu, Justin Ryan L. Tan, Hang Bin Jin, Quifeng Lou, Ka Shing Cheung, Jianfeng Yang, Xiaofeng Zhang

**Affiliations:** 1 Department of Gastroenterology, Hangzhou First Peopleʼs Hospital, Hangzhou, China; 2 Section of Gastroenterology, Chinese General Hospital and Medical Center, Manila, Philippines; 3 444333 Department of Medicine, The University of Hong Kong-Shenzhen Hospital, Shenzhen, China; 4 26473 Queen Mary Hospital, Hong Kong, Hong Kong

A 23-year-old woman presented with a 1-week history of abdominal pain radiating to the back. The pain was not relieved by proton pump inhibitors. Contrast-enhanced abdominal computed tomography scan demonstrated a round, well-defined, hypodense lesion within the pancreatic body ( Fig. 1 ). On endoscopic ultrasound (EUS) elastography there was a 2 × 2.5-cm hypoechoic mass that was predominantly hard (blue) with dispersed heterogeneous soft (green) areas ( Fig. 2 ). Contrast-enhanced harmonic endoscopic ultrasound (CH-EUS) revealed hyperechoic solid granular components interspersed with small anechoic regions within the pancreatic mass ( Fig. 3 ). These characteristics were typical of the “alveolus nest sign” and were present during the arterial and venous phases of CH-EUS ( Video 1 ). EUS-guided fine-needle biopsy with subsequent histopathologic examination ( Fig. 4 ) and immunohistochemical analysis ( Fig. 5 ) yielded a definitive diagnosis of solid pseudopapillary neoplasm (SPN). The patient underwent distal pancreatectomy for definitive treatment.

**Fig. 1:**
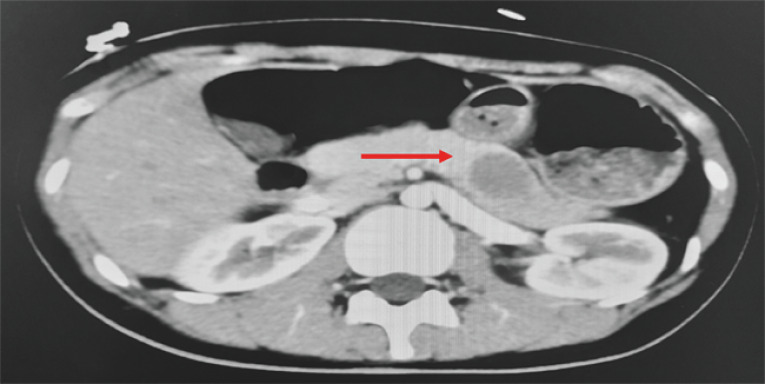
Contrast-enhanced computed tomography scan revealed a round, well-defined, hypodense lesion (red arrow) within the pancreatic body.

**Fig. 2:**
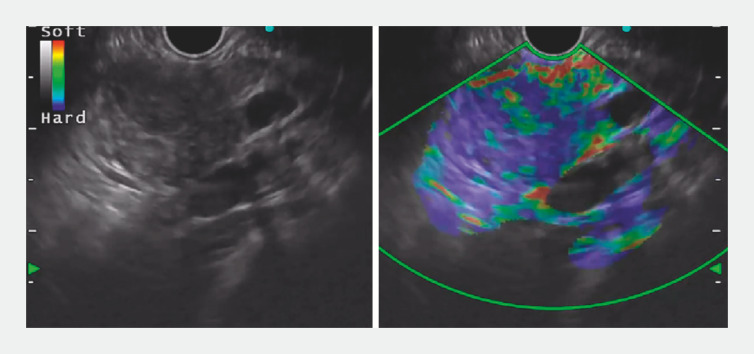
Endoscopic ultrasound elastography revealed a 2 × 2.5-cm hypoechoic mass that was predominantly hard (blue) with dispersed heterogeneous soft (green) areas.

**Fig. 3:**
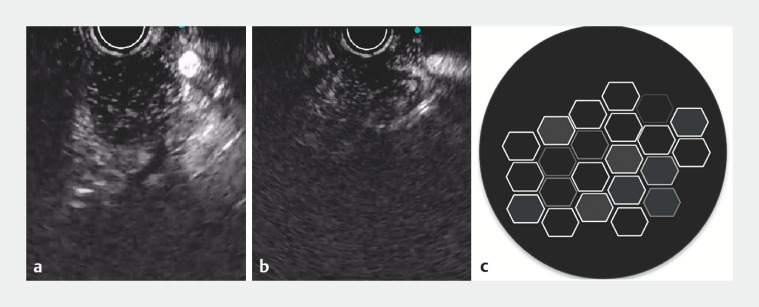
Contrast-enhanced harmonic endoscopic ultrasound revealed hyperechoic solid granular components interspersed with small anechoic regions within the pancreatic mass. **a** Arterial phase. **b** Venous phase. **c** Visual representation of the alveolus nest sign.

**Fig. 4:**
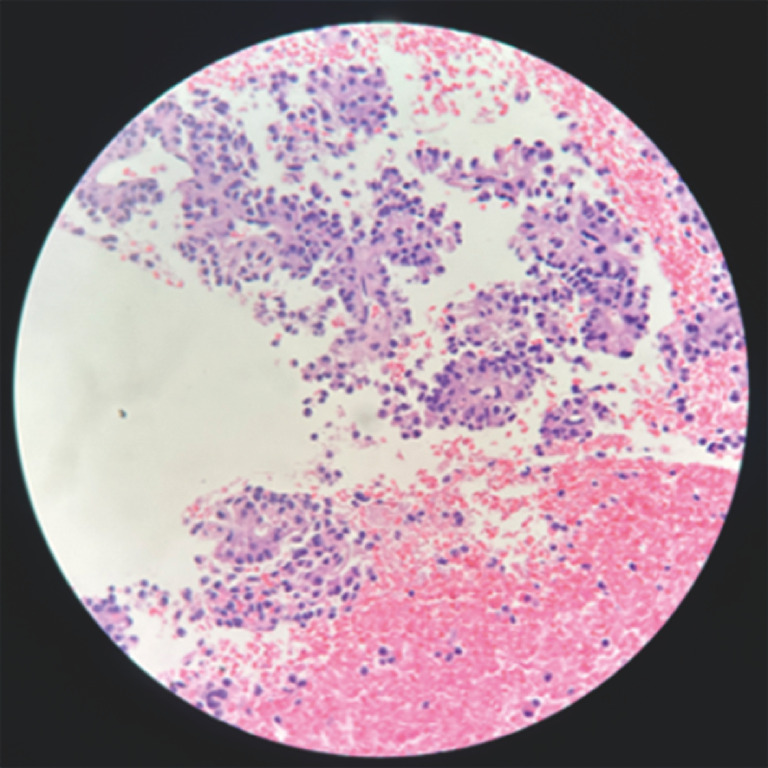
Histopathology specimen showed scattered epithelioid cells with clear or eosinophilic cytoplasm arranged in sheets or papillary shapes.

**Fig. 5:**
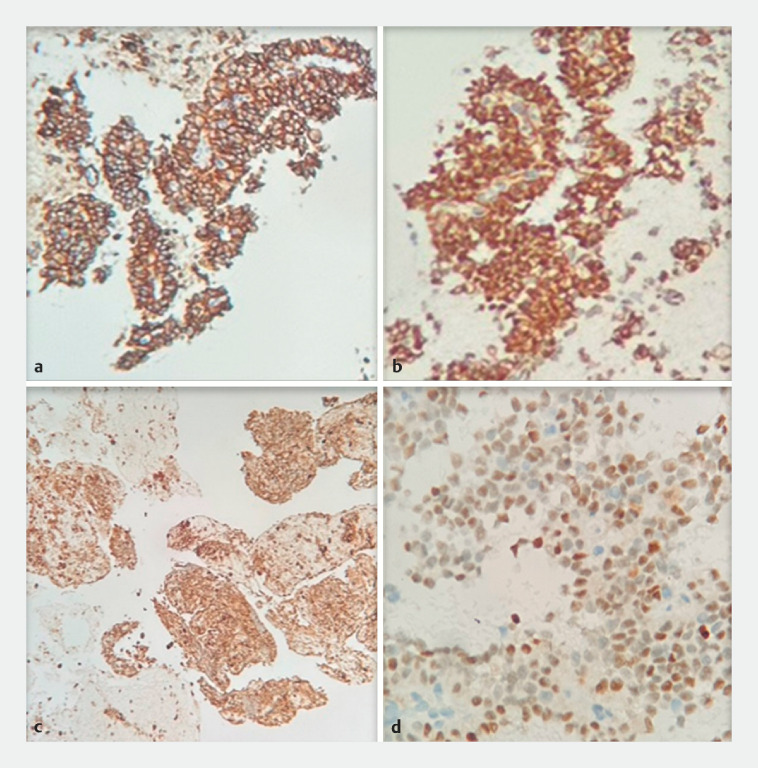
Immunohistochemical tests showed the following positive results. **a** CD56. **b** β-catenin. **c** α-1-antitrypsin. **d** Progesterone receptor.

The alveolus nest sign during the arterial and venous phases of contrast-enhanced harmonic endoscopic ultrasound.Video 1

SPN is an uncommon pancreatic neoplasm exhibiting low malignant potential. It predominantly affects young females and is characterized by indolent behavior, with an excellent long-term prognosis for the majority of patients 1 2 3 4 . CH-EUS examination may reveal characteristic features suggestive of SPN pathology, including the presence of an intralesional pseudopapillary architecture or intratumoral clefting, also termed the “alveolus nest sign” 1 2 . On CH-EUS, the alveolus nest sign is seen as a region with isoechogenicity to hyperechogenicity, containing a solid and granular component. This component is interspersed with multiple small anechoic areas of varying sizes. These anechoic regions become evident on CH-EUS typically 40 seconds after contrast injection. The presence of this sign at any time within 40 seconds to 5 minutes post-contrast administration is considered characteristic of the alveolus nest sign 1 2 . CH-EUS is useful in differentiating SPN of the pancreas from other pancreatic tumors. Alveolus nest sign is a characteristic feature of SPN on CH-EUS.

Endoscopy_UCTN_Code_CCL_1AF_2AZ

## Notes

Endoscopy E-Videos https://eref.thieme.de/e-videos
*E-Videos* is an open access online section of the journal *Endoscopy* , reporting on interesting cases and new techniques in gastroenterological endoscopy. All papers include a high-quality video and are published with a Creative Commons CC-BY license. Endoscopy E-Videos qualify for HINARI discounts and waivers and eligibility is automatically checked during the submission process. We grant 100% waivers to articles whose corresponding authors are based in Group A countries and 50% waivers to those who are based in Group B countries as classified by Research4Life (see: https://www.research4life.org/access/eligibility/ ). This section has its own submission website at https://mc.manuscriptcentral.com/e-videos .
